# Improving prioritization processes for clinical practice guidelines: new methods and an evaluation from the National Heart Foundation of Australia

**DOI:** 10.1186/s12961-022-00953-9

**Published:** 2023-04-05

**Authors:** Brooke Atkins, Tom Briffa, Cia Connell, Amanda K. Buttery, Garry L. R. Jennings

**Affiliations:** 1 National Heart Foundation of Australia, 2/850 Collins Street, Melbourne, VIC 3008 Australia; 2 School of Population and Global Health, University of Western Australia, Clifton Street Building, Clifton St, Nedlands, WA 6009 Australia; 3 University of Sydney, Sydney, NSW 2006 Australia

**Keywords:** Priority-setting, Clinical practice guidelines

## Abstract

**Background:**

Releasing timely and relevant clinical guidelines is challenging for organizations globally. Priority-setting is crucial, as guideline development is resource-intensive. Our aim, as a national organization responsible for developing cardiovascular clinical guidelines, was to develop a method for generating and prioritizing topics for future clinical guideline development in areas where guidance was most needed.

**Methods:**

Several novel processes were developed, adopted and evaluated, including (1) initial public consultation for health professionals and the general public to generate topics; (2) thematic and qualitative analysis, according to the International Classification of Diseases (ICD-11), to aggregate topics; (3) adapting a criteria-based matrix tool to prioritize topics; (4) achieving consensus through a modified-nominal group technique and voting on priorities; and (5) process evaluation via survey of end-users. The latter comprised the organization’s Expert Committee of 12 members with expertise across cardiology and public health, including two citizen representatives.

**Results:**

Topics (*n* = 405; reduced to *n* = 278 when duplicates removed) were identified from public consultation responses (*n* = 107 respondents). Thematic analysis synthesized 127 topics that were then categorized into 37 themes using ICD-11 codes. Exclusion criteria were applied (*n* = 32 themes omitted), resulting in five short-listed topics: (1) congenital heart disease, (2) valvular heart disease, (3) hypercholesterolaemia, (4) hypertension and (5) ischaemic heart diseases and diseases of the coronary artery. The Expert Committee applied the prioritization matrix to all five short-listed topics during a consensus meeting and voted to prioritize topics. Unanimous consensus was reached for the topic voted the highest priority: ischaemic heart disease and diseases of the coronary arteries, resulting in the decision to update the organization’s 2016 clinical guidelines for acute coronary syndromes. Evaluation indicated that initial public consultation was highly valued by the Expert Committee, and the matrix tool was easy to use and improved transparency in priority-setting.

**Conclusion:**

Developing a multistage, systematic process, incorporating public consultation and an international classification system led to improved transparency in our clinical guideline priority-setting processes and that topics chosen would have the greatest impact on health outcomes. These methods are potentially applicable to other national and international organizations responsible for developing clinical guidelines.

**Supplementary Information:**

The online version contains supplementary material available at 10.1186/s12961-022-00953-9.

## Background

Ensuring clinical guidelines are contemporary with evolving evidence and practice standards is a global challenge [1]. Formulating guidelines may take between 18 and 30 months to complete [2] and therefore generating and prioritizing topics is a critical first step in the development process. The importance of priority-setting is recognized by many guideline developers and international organizations in the United States [3], United Kingdom [4, 5] and Europe [6], but is frequently the least transparent step in publishing clinical guidelines.

Globally, many organizations involved in guideline development including WHO [7] and the National Institute for Health and Care Excellence [5] report using specific criteria or questions to prioritize the topics selected for clinical guideline development and updates. While checklists and criteria exist for priority-setting, the processes for applying these criteria and making decisions are often not explicitly documented [1, 6, 8, 9], which limits transparency and the ability to generalize their processes. Failure to document key information about how guidelines are developed, including how and why topics are chosen, may also impact implementation efforts [2].

There are a variety of approaches for priority-setting available, and the James Lind Alliance, an initiative established in 2004 in the United Kingdom, provides step-by-step guidance on how to set up priority-setting partnerships by bringing together patients, carers and clinicians to identify the top 10 unanswered questions or evidence uncertainties, particularly for setting research priorities [4]. However, some of the evidence available around priority-setting methods for research are challenging to adapt and apply to clinical guideline development [4, 10, 11].

The National Health and Medical Research Council (NHMRC) is the organization largely responsible for clinical practice guidelines in Australia. The NHMRC have published the 2016 NHMRC Standards for Guidelines [12] and a suite of tools for guidelines developers including self-contained peer-reviewed modules on the NHMRC website called the Guidelines for Guidelines Handbook [13]. However, processes about priority-setting including topic generation and selection are lacking from the guidance, which largely assumes that topics have already been selected for guideline development.

The National Heart Foundation of Australia is an independent, not-for-profit organization that funds cardiovascular research and works to improve heart disease prevention, detection and support for all Australians [14]. Alongside funding research, the organization, in partnership with others, develops clinical practice guidelines and position statements for healthcare professionals. Our primary aim was to determine areas within cardiovascular disease where clinical guidelines would have the greatest impact on health outcomes and where guidance was most needed. Our secondary aim was to improve transparency and certainty in our methods for generating and prioritizing topics for clinical guideline development and updates.

## Methods

### Study design

We undertook a quality improvement initiative to update our methods for priority-setting for clinical guideline development. This involved conducting an online public consultation for health professionals and the public to generate topics for guidelines, an iterative process to refine topics through thematic analysis and categorization using the current WHO International Statistical Classification of Diseases and Related Health Problems (ICD) [15], developing a criteria-based matrix for prioritizing topics and adopting established consensus techniques to decide on priorities. Each of these is steps is described below. The Standards for Quality Improvement Reporting Excellence (SQUIRE) checklist was used for reporting [16] and is presented in Additional file 1.

### Expert Committee

The organization’s internal governance structures include an established Expert Committee who provide guidance on clinical issues including the development and implementation of clinical guidelines and position statements. The Expert Committee comprises 12 members with expertise across cardiology, public health and epidemiology, and includes two people with lived experience of heart conditions. The Expert Committee approved, developed and implemented this quality improvement initiative with support from the organization’s clinical and evaluation teams. Three members of the Expert Committee (the Committee Chair, Deputy Chair and the organization’s Chief Medical Advisor) were closely involved in study design and processes, and hereafter are referred to as the Expert Subcommittee.

### Public consultation to generate topics

Public consultation was the first step to generate topics for selection. A five-item online public consultation survey (Table 1) was developed with approval from the Expert Subcommittee. The public consultation was held for a period of 7 weeks, between 22 November 2019 and 10 January 2020, aligning with national recommendations that a public consultation is open for a minimum 30-day period [17]. The online survey was developed using the Typeform^®^ software platform [18]. The survey included a privacy statement, and data collection was anonymous. The distribution methods for the public consultation survey are presented in Table 2. People were provided with a description as to the purpose of the survey and a direct link to share with others; thus, a snowball recruitment technique was also part of the survey distribution approach. Members of the organization’s committees comprised people both internal (employees, volunteers and committee members) and external to the organization.

**Table 1:** Survey questions included in public consultation

1. I am providing information as a: (A) Member of the public (B) Healthcare professional (13 categories of responses available)
2. What cardiovascular disease theme(s) do you think are a priority for the Heart Foundation?
3. Why is this clinical theme of significance to the Australian community?
*(e.g. is there a significant burden of disease, prevalence, or economic impact? Is there opportunity to reduce inequity?)*
4. Why is information and advice needed on this theme currently?
*(e.g. is there new, emerging or rapidly changing evidence or new care options? Is there complexity, controversy or uncertainty about themes and treatment?)*
5. What area(s) of care require information and advice about this theme?
*(e.g. prevention, screening, diagnosis, treatment, management)*

**Table 2:** Distribution of public consultation survey

Method	Date promoted	Number of recipients	Type of stakeholder
All-staff announcement via internal newsletter	27/11/2019	272 staff members	Internal
Direct electronic mail to all staff	22/11/2019	272 staff members	Internal
Organization’s advisory committees	22/11/2019 07/01/2020	40 members	Internal and external
Organization’s Twitter account	25/11/2019 07/01/2020	Approximately 24,800 subscribed	External
Newsletter to healthcare professionals	22/11/2019	Approximately 20,000 heath care professionals subscribed	External
Direct electronic mail to health and consumer organizations	22/11/2019	69 unique IP addresses	External
Organization’s webpage	22/11/2019–10/01/2020	There were 43,825 webpage views during the period the survey was promoted on the organization’s website	External

### Data analysis and short-listing of topics

An Excel spreadsheet containing responses was downloaded from the survey software platform at the close of the public consultation. Data were extracted from responses using descriptive coding to identify topics (e.g. valve disease, stroke, exercise). Topics were then categorized (e.g. cardiac arrhythmia, hypertensive disease) using 118 codes from the International Classification of Diseases 11th Revision (ICD-11) [15]. Any remaining topics that were unable to be classified using the ICD-11 were grouped into broader themes (e.g. obesity, cardiovascular disease prevention, supportive care). All themes were then grouped into four broad categories: (i) diseases of the circulatory system, (ii) other diseases related to cardiovascular disease, (iii) risk factors and prevention strategies, and (iv) general themes for guideline development such as patient support and secondary prevention. Two reviewers (BA and CC) were involved in all phases of data analysis. This iterative process of drafting and revising topics, themes and categories resulted in consensus of the two reviewers. It was planned that disagreements between the two reviewers would be resolved by an Expert Subcommittee member (third reviewer), but this was not required. The final categories and results (Additional file 2) were reported to the Expert Subcommittee for validation. All agreed with the categorization of topics without further refinement.

A four-item set of exclusion criteria was developed (Table 3) and applied to short-listed topics. A 1-hour teleconference was held with the Expert Subcommittee on the 4th of March 2020 to apply the exclusion criteria to create a finalized list of short-listed topics through consensus.

**Table 3:** Exclusion criteria applied to categorized topics

1. Topic is nonclinical^a^/public health^b^/prevention^c^
2. Topic is a general theme—not a topic for clinical guideline development^d^
3. Topic is not limited to heart disease 3a. Topic is cardiovascular disease, but not specific to heart
3b. Topic is related to cardiovascular disease, but not specific to cardiovascular disease
4. Guidelines published, or published guidelines funded by the National Heart Foundation of Australia within the last 2 years

^a^Nonclinical: topics not classified using the IDC-11 code e.g. “urban environments”

^b^Public health topics not specific to cardiovascular disease e.g. “obesity”

^c^Prevention topics related to prevention strategies or risk factors e.g. “smoking cessation”

^d^Topics classified as general themes or areas of guidance routinely included in clinical guidelines, but are a topic for clinical guidelines, e.g. “medication management, “patient support”

### Development of criteria-based matrix tool to prioritize topics

We drew upon criteria used by other guideline developers [5–7, 19] and criteria identified in published literature [1, 5, 8, 9, 11, 20] to form common categories to prioritize topics (e.g. prevalence of a disease, healthcare expenditure). We followed an iterative process of consolidating and revising criteria for the proposed matrix and compared this to the organization’s existing matrix tool. This process involved drafting of the matrix tool by one author (BA), and another author (CC) revising the draft matrix tool to further consolidate criteria and improve clarity including merging and modifying the criteria. The matrix tool was piloted with a subset of short-listed topics. Further refinement of the tool was achieved by the Expert Subcommittee until consensus was reached and the tool was finalized. The newly developed matrix tool (see Additional file 3) had five domains and considerations listed within each. This was presented in a descriptive version of the tool (see Additional file 4). Criteria within the matrix were not ranked or weighted, as evidence suggests that there is large variance in individuals’ ratings of the importance of criteria and no difference in topic selection when weighing the criteria [21].

### Consensus meeting to decide on priorities

A face-to-face consensus meeting with the Expert Committee was planned to apply the matrix tool to prioritize topics. However, a videoconference was held due to COVID-19 travel restrictions (total attendees *n* = 16: Expert Committee members *n* = 11; *n* = 5 staff members from the organization).

Prior to meeting, all attendees were sent the following documents: the organization’s strategic plan, a description of the literature used to develop the matrix tool, the results of the public consultation responses in a report format, the final summary report of the categorized topics, the exclusion and inclusion list developed as part of the process, the matrix tool and a description of how to use the tool, and an economic report for short-listed topics prepared by a health economist within the organization. The economic report included data and statistics on incidence, prevalence, mortality, hospitalization, burden of disease and healthcare expenditure for each topic to support matrix criteria. The two people with lived experience of heart disease received additional information via telephone prior to the meeting including a description of the planned process to prioritize topics using the matrix tool and voting procedures. They were both provided with a glossary of clinical terms in plain language as a supplementary document prior to the meeting.

During the meeting the prioritization process was described and where available, additional data were presented on emerging evidence, gaps in knowledge on these topics, and variance in care and equity considerations. Discussions and all decisions made by the Expert Committee were documented on matrix tools. The chair of the meeting invited members with expertise on a topic to lead discussions for that topic, such as a member with specialist expertise in lipidology led the discussion on hypercholesterolaemia. A structured process of checking that each criterion had been addressed by the committee before moving to the next topic was adopted during the meeting.

A consensus-based approach using the modified-nominal group technique [22] was used to generate and refine discussion within the Expert Committee and prioritize short-listed topics. An online poll was created for attendees to anonymously vote on topics using the software Poll Everywhere^®^ [23]. During the videoconference, the Expert Committee agreed to vote verbally, and each Expert Committee member (*n* = 11) listed topics in order of their priority.

### Evaluation survey

A survey was used to evaluate the effectiveness and acceptance of the new prioritization process. The organization’s evaluation unit—a group independent of those involved in the design and implementation of the new prioritization process—designed, distributed and analysed the survey results. The 23-item online anonymized evaluation survey was developed using Typeform^®^ software [18] (see Additional file 5). The survey was sent to all attendees of the videoconference (*n* = 16) on 2 April 2020, followed by two reminders. The survey results were analysed and presented as a report and distributed to all Expert Committee members to review.

## Results

### Public consultation

The initial online public consultation resulted in 405 potential topic suggestions from 107 people. Most people responding to the survey were healthcare professionals or researchers (66%; 71/107) or members of the public (34%; 36/107) (Table 4). Responses were received from across the country, including all six states and two territories. Healthcare professionals and researchers were given the option to provide their workplace, and 40 different organizations including hospitals, state health services, universities and research institutions were represented.

**Table 4:** Public consultation respondents by occupational categories and frequencies of responses

Type of respondent (mutually exclusive categories)	Frequencies of responses (*n* = 107)
Member of the public	36
Pharmacist	15
Nurse	14
Allied health	11
Healthcare professional–other	11
Healthcare researcher	10
Cardiologist	5
General practitioner	2
Cardiac surgeon	1
Junior doctor	1
Nurse practitioner	1

### Data analysis and short-listing of topics

There were 405 topics identified from public consultation that were categorized to 127 themes, then mapped against 118 ICD-11 codes from 11 chapters [15], resulting in 37 themed topics across four broad areas: diseases of the circulatory system, other diseases related to cardiovascular disease, risk factors and general health themes. Ischaemic heart diseases and diseases of the coronary artery have separate ICD-11 codes; however, it was decided by the Executive Subcommittee to group these as one topic, as guidance in this area would cover both topics. Application of the exclusion criteria resulted in five short-listed themes: (i) congenital heart disease, (ii) valvular heart disease, (iii) hypercholesterolaemia, (iv) hypertension and (v) ischaemic heart diseases and disease of the coronary artery.

### Application of the criteria-based matrix tool to prioritize topics

For each short-listed topic, a separate matrix tool was used to document discussions and decisions and served as minutes of the meeting. The completed matrix tool for ischaemic heart diseases and diseases of the coronary artery is provided as an example in Table 5. Evaluation indicated that presenting topics with supporting data for criteria with the matrix helped structure information to facilitate decision-making. Following the meeting, completed matrix tools were reviewed by all members of the Expert Committee. Minor changes were made following review, and all matrix tools were accepted as accurate and final by the Expert Committee at a subsequent meeting on 16 April 2020.

**Table 5:** Completed matrix tool for ischaemic heart diseases and diseases of the coronary artery

(1) Impact of disease	(2) Potential to impact health outcomes	(3) Organization’s strategy	(4) Need from our community	(5) Relevance to broad range of healthcare professionals	(6) Evidence base
- Burden of disease, in terms of mortality, incidence or prevalence of disease - Economic impact and costs *Does guideline development in this area have the potential to ****impact many people**** affected by this disease?*	- Significantly improve health outcomes/promote health/reduce inequalities - Significant or unexplained variation in clinical practice - Feasibility of implementing a guideline - Reduce avoidable mortality or morbidity *Would a guideline on this topic ****feasibly address or impact clinical practice and health outcomes and reduce variance in care?***	- Relevance to our 2018–2020 Strategy *Would a guideline on this topic be ****on strategy for our organization?***	- Topic representation during clinical themes public consultation by healthcare professionals and consumers - Misconception about topic within the general community *Is there demonstrated ****feedback**** from our community that a guideline on this topic is ****needed****?*	- Relevance of topic to healthcare workers with a diverse level of expertise where most of the care is delivered by nonexperts *Is care in this clinical area significantly delivered by ****nonexperts?***	- New, emerging or rapidly changing evidence or new care options - Complexity, controversy or uncertainty about topics and treatment - Level and quality of current evidence on topic - Published guidelines or guidelines funded by our organization within the past 2 years already exist *Would a guideline on this have a ****strong evidence base**** or address any controversy in the interpretation of the current evidence base?*
Some deaths are the first presentation—sudden cardiac death due to coronary heart disease. This disease is daily work for general practice. Enormous benefit in developing guidelines in this area with a huge scope that may span a number of guidelines. Huge impact if applied well. Scope primary and secondary care. Suggest update guidelines for primary prevention—absolute risk and hypertension in one document If prevalence data include risk factors, this will include a lot more people Noted ischaemic heart disease and coronary artery disease are used interchangeably; however, the latter does not cover stroke or peripheral vascular disease and is more specific to heart disease	Decide whether primary, subclinical or manifest disease Mental health is an area of inequality and there is high prevalence in this group. Psychiatric disease and coronary artery disease need specialized care Early-onset cognitive decline noted as another special population	Noted this is identified in our current strategy	Guidance for targeted use of aspirin needed for individuals who will truly benefit Medication adherence, deprescribing and shared decision-making identified as areas requiring guidance/support Misconception around statins and medication in general in patients Guidelines on when medication can safely be withdrawn needed Discussed intellectual commentary for general practitioners about deprescribing, duration of therapies and the evidence base around this or guidelines to include this and discuss the evidence base/impacts to align a common approach	Some components of specialist input, largely general practice	Current guidelines noted—hypertension, acute coronary syndromes and absolute risk Noted absolute risk guidelines are being updated—will be discussing in the next 4–6 weeks, this will impact scope depending on what is covered by these guidelines Update guidelines needed for primary prevention (like New Zealand did) Discussion on living guidelines and Magic app—how will we update guidelines we already have?

### Consensus meeting to decide on priorities

Expert Committee members (*n* = 11) voted on topics verbally during the videoconference, resulting in short-listed topics being prioritized with 1 being the highest priority and 5 being the lowest priority (Table 6). All members were in agreement with the topic that was voted the highest priority: ischaemic heart diseases and diseases of the coronary artery. All respondents agreed that the purpose of the meeting was clear, and that the meeting duration (5.5 hours) was acceptable. Most respondents felt they were able to share their opinions. Feedback from the evaluation to improve future processes included providing explicit information on dates and timelines when a clinical guideline was last reviewed.

**Table 6:** Ranked voting result of short-listed topics during consensus meeting

Short-listed topics	Priorities (scores from each Expert Committee member *n* = 11)											Sum of raw scores^a^	Ranked priority
	1	2	3	4	5	6	7	8	9	10	11		
Ischaemic heart diseases and diseases of the coronary artery	1	1	1	1	1	1	1	1	1	1	1	55	#1
Hypercholesterolaemia	3	2	3	2	3	3	2	5	3	4	2	35	#2
Hypertension	2	3	2	5	2	2	3	4	5	2	5	31	#3
Valvular heart disease	4	4	4	3	4	4	4	2	2	3	4	27	#4
Congenital heart disease	5	5	5	4	5	5	5	3	4	5	3	17	#5

^a^Sum of raw scores: Points allocated to each short-listed topic using raw scores (top priority = five points, fifth priority = one point)

### Process evaluation survey results

The results from the evaluation survey showed that respondents valued public consultation highly as the first step in the process. There were varied responses regarding the quality of topics generated from the public consultation; however, most respondents agreed that public consultation should continue as the first step in the prioritization process. Common themes from individual respondents were that public consultation was best practice and vital to ensuring the organization remains relevant to all stakeholders. One Expert Committee member reported that public consultation “is vital to ensure the organization remains relevant to all stakeholders in the community”, and another that public consultation is an “important reality check for clinicians with specialist knowledge”. Overall, respondents agreed that the new processes were effective, systematic and easy to follow. Five respondents reported they would have preferred a face-to-face meeting rather than a videoconference, but acknowledged that it was not feasible given the restrictions on nonessential travel in Australia at the time.

## Discussion

The first step in our new multistage process was to generate topics for guideline development through public consultation involving the public and health professionals. Only topics that were generated from public consultation were considered to ensure topics were in areas where guidance was most needed from the wider community. The ICD codes were used to categorize topics. The Expert Committee used the new matrix tool to decide upon priorities. We explicitly documented how decisions were made, and by whom. Evaluation demonstrated that this increased the transparency and certainty of our methods.

Public consultation to generate topics is increasingly recognized as an important component of clinical guideline development [9, 17, 24, 25]. Additionally, many clinical guideline developers recommend health consumer involvement as part of the broader guideline development process [5, 7, 24]. However, many do not explicitly document methods for health consumer and patient involvement [1, 6, 8, 9, 26]. Evaluation of our process demonstrated that public consultation was perceived as an important “reality check” for clinicians by Expert Committee members, and most agreed that public consultation should continue to be used to generate topics for clinical guideline development. Comparatively, the American Heart Association describe their processes as involving a task force comprising senior, well-respected individuals with a variety of expertise to choose individual topics for guideline development and do not mention patient or health consumer involvement [3]. A study involving two parallel guideline development groups, one with and one without patient representatives, found that patient involvement led to the inclusion of patient-relevant topics [27]. Patient representatives helped identify issues that may be overlooked by medical professionals and helped select patient-relevant outcomes [27]. The two Expert Committee members with lived experience of heart disease contributed to ensuring that patient-specific issues were considered, such as access to care and services, as one member lived in a rural area and primarily sought care from a general practitioner rather than travelling a long distance to see a specialist. This informed discussions to prioritize topics and support our aim to develop clinical guidelines in areas where guidance was most needed. A barrier to adapting existing criteria and checklists proposed by others is that they were often not specific to guideline development [1, 4, 10, 11]. Additionally, many processes, while comprehensive, propose numerous items to consider as part of their criteria and often there is duplication of criteria. The McMaster Group in Canada, for their project Guidelines 2.0, developed a checklist that includes 18 topics and 146 items [1]. The checklist is broad and therefore potentially has wider applicability, including public health and policy guidelines; however, it may not be suitable or feasible in all settings given its length [1]. We developed a matrix tool through refining, merging and synthesizing criteria proposed by other organizations and by guideline developers to produce a practical tool that can help prioritization. In Australia, the Australian Commission on Safety and Quality in Health Care (the Commission) leads and coordinates national improvements in the safety and quality of healthcare [28]. The Commission and the NHMRC have proposed a national framework to promote the efficient development of clinical practice guidelines [8]. All criteria in this framework [8] were incorporated into the priority-setting matrix tool. Evaluation found that most of the Expert Committee felt that the new matrix tool contained all the important criteria needed for decision-making. As our organization develops clinical guidelines in cardiology, we applied exclusion criteria to short-list topics only related to cardiovascular disease, and included additional criteria in the matrix tool to assess a topic’s alignment with the Heart Foundation’s strategy [28]. Short-listing topics improved the feasibility of providing additional data for each topic to support the application of the matrix and ensured that the census meeting held with the Expert Committee was an acceptable duration. Many organizations that focus on specific health conditions, such as cardiovascular, stroke or other neurological conditions, could further refine and adapt the exclusion criteria and matrix tool to align guideline priorities with their organization’s missions and values and the needs of the communities they serve.

In 2014, the NHMRC analysed 515 clinical practice guidelines on their database and reported that there were serious and systemic problems with the way guidelines were developed in Australia and called for new methods to promote more transparent documentation of guideline development processes to improve the quality of guidelines [2]. This recommendation applies to topic generation and the prioritization of topics for selection, as this is the first step in guideline development. Consensus-based methods have been established as an effective and accepted way to decide upon priorities [22]. Verbal ranking of topics was performed to decide upon priorities by each Expert Committee member. The matrix criteria were used as guidance for discussion to ensure that all important aspects were considered. Voting using the modified nominal group technique enabled each member to have an equal vote, which was important given the Expert Committee comprised members with knowledge and expertise in different areas and included two members with lived experience of heart disease. The evaluation survey found Expert Committee members found this process transparent, systematic and effective.

While additional and economic data were prepared for all short-listed topics, it was not possible to systematically report this due to the variation between data collection methods and availability between jurisdictions within Australia. For example, at the time this initiative was conducted, the number of people with congenital heart disease was not collected in a uniform way by jurisdictions, or through a national clinical registry, and therefore the true prevalence of people with congenital heart disease in Australia was unknown. Expert Committee members with known expertise on a topic led discussions, which possibly influenced the nature and depth of discussion. However, for each topic, every panel number was invited by the chair to contribute to the discussion. It is not possible to exclude bias when applying the prioritization matrix, as topics with more data and in-depth discussion may have ranked higher among members. However, this bias was minimized by transparent reporting on known gaps in data and knowledge, and through the use of the multifactorial criteria, as additional information informed the discussion of two of the five criteria in the matrix. Unanimous consensus was achieved for the topic voted highest priority, which may indicate that inconsistent data availability and the topic-specific expertise of individual members is unlikely to have impacted the outcome of this process.

There are several contextual factors unique to the development and application of this process for prioritizing topics for clinical guideline development. The skill set of the Expert Committee, which although common among clinical guideline developers [3, 5–7] were unique to the organization and included experts in cardiology, public health and epidemiology and those with lived experience of cardiac conditions, likely influenced results. The chair of the consensus meeting, who was also a member of the Expert Subcommittee, was experienced in the role of chairing meetings and group consensus methods. This experience is likely to have positively influenced application of the matrix tool and overall results of the improvement initiative. Additionally, the organization’s health economist assisted in preparing economic data and guidance on how this data could be used in combination with the prioritization process and the matrix. Furthermore, as a national organization producing numerous guidelines, we were able to promote the public consultation through our large networks of health professionals and community. We appreciate that other guideline developers, such as professional associations and agencies, and those that focus on a single specific disease may be limited in the use of public consultation to generate an adequate amount of topics, depending on the size of their networks.

## Strengths and limitations

There are several strengths of our study. Firstly, it responds to the NHMRC and the Australian Commission on Safety and Quality in Health Care’s call for more transparent criteria and processes for priority-setting for clinical guideline development [2, 8]. We undertook thematic analysis of data extracted from public consultation results and used ICD-11 codes to map topics to prioritize clinical guideline topics for development. To our knowledge, this is the first time that ICD codes, especially version 11, have been used to map and categorize domains for guideline development. Using standardized internationally recognized classification potentially increases the applicability of the prioritization process for other guideline developers. Limitations to our study include a lower-than-expected response rate to the public consultation. The public consultation process occurred over the main national holiday period between December 2020 and January 2021. The seasonal timing of the survey was likely to have influenced the response rate, despite the consultation period being extended. However, over 400 topics were identified from the 107 responses, highlighting that those who did respond were engaged with the topic of cardiovascular health and that survey questions were effective in generating topics. Another limitation of this study is that not all members of the Expert Committee completed the evaluation survey, and positive response bias may have occurred.

## Implications and next steps

The processes outlined in this quality improvement initiative, including application of the matrix tool, could easily be adopted and adapted for other clinical guideline developers. Furthermore, the process by which we supplied economic data alongside the matrix criteria was valued by decision-makers and could be reproduced for other diseases and priority-setting activities. Public consultation was the first step in our new process and was highly valued by the Expert Committee. Both end-users and the public should be involved in priority-setting for clinical guideline development, including public consultation to generate topics. This was an important first step in our process, as it highlighted areas where guidance was most needed. Prioritizing clinical guidelines for other health topics unrelated to cardiovascular disease may also be suitable using ICD codes, for example, for neurological or endocrine disorders. By documenting how decisions were made, and by whom, we have increased the transparency of our approach and enabled other organizations to apply our methods. We recommend that other guideline developers document decision-making and make this publicly available to improve transparency. Further methodological research is needed to refine this process and assess the effectiveness and acceptability for other guideline developers, including in areas other than cardiovascular disease.

## Conclusions

We have described our approach for generating and prioritizing topics for clinical guideline development, including initial public consultation, the development of a new matrix tool, adopting established consensus-based methods for voting on final priorities, and evaluation of the process. These methods resulted in the prioritization of topics and selection of a topic for guideline development in an area that demonstrated the most need with the potential to improve health outcomes. By explicitly documenting our methods, including decision-making, we have improved the transparency of our guideline development process. These methods can be adopted and adapted by other clinical guideline developers.

## Contributions to the literature

- Clinical practice guidelines take considerable effort, time and resources to develop. Numerous checklists and methods for selecting which topics to prioritize for clinical guideline development and updates are available; however, how decisions are made, and by whom, are poorly understood.
- We developed a new process for prioritizing topics for clinical guideline development that involved both the public and health professionals to generate topics.
- We used WHO’s ICD codes to categorize topics. We developed a new tool to prioritize these topics. We explicitly documented how decisions were made, and by whom, to increase transparency.
- By documenting our methods, our process and tools can be adopted and adapted by international guideline developers not only for cardiovascular diseases, but other conditions.

## Supplementary Information

**Additional file 1.** SQUIRE checklist. Description of data: Completed SQUIRE reporting checklist.

**Additional file 2.** Topics categorized using ICD-11 codes. Topics from public consultation extracted and categorized according to the International Classification of Diseases 11th revision, including topic keywords and frequency the topic was suggested in public consult responses.

**Additional file 3. **Matrix tool. This matrix tool with five domains was used by the Expert Committee during the consensus meeting to assist in prioritizing short-listed topics.

**Additional file 4.** Descriptive version of the matrix tool. Expanded version of the matrix tool listing considerations within each domain. This was provided to the Expert Committee before the consensus meeting.

**Additional file 5.** 23-item online survey questionnaire. The 23-item online anonymized evaluation survey sent to Expert Committee members after the consensus meeting.

## Data Availability

Not applicable to this manuscript, as no datasets were generated or analysed during the current study.

## Abbreviation

ICD-11: International Classification of Diseases 11th Revision
