# Supplementary material for: Improving prioritization processes for clinical practice guidelines: new methods and an evaluation from the National Heart Foundation of Australia
Source: Health Res Policy Syst. 2023 Apr 5;21:26. doi: 10.1186/s12961-022-00953-9 (PMC10075165; doi:10.1186/s12961-022-00953-9)
Supplement: Supplementary file 1 — Additional file 1. SQUIRE checklist. Description of data: Completed SQUIRE reporting checklist. [file 12961_2022_953_MOESM1_ESM.docx]

| **Research and reporting methodology** |  | |  |
| --- | --- | --- | --- |
| Revised **Standards for QUality Improvement Reporting Excellence** (**SQUIRE 2.0**) publication guidelines |  | |  |
|  |  | |  |
| **Notes to authors** |  | |  |
| ▸ The SQUIRE guidelines provide a framework for reporting new knowledge about how to improve healthcare. |  | |  |
| ▸ The SQUIRE guidelines are intended for reports that describe system level work to improve the quality, safety and value of healthcare, and used methods to establish that observed outcomes were due to the intervention(s). |  | |  |
| ▸ A range of approaches exists for improving healthcare. SQUIRE may be adapted for reporting any of these. |  | |  |
| ▸ Authors should consider every SQUIRE item, but it may be inappropriate or unnecessary to include every SQUIRE element in a particular manuscript. |  | |  |
| ▸ The SQUIRE glossary contains definitions of many of the key words in SQUIRE. |  | |  |
| ▸ The explanation and elaboration document provides specific examples of well-written SQUIRE items and an in-depth explanation of each item. |  | |  |
| ▸ Please cite SQUIRE when it is used to write a manuscript. |  | |  |
|  |  | |  |
| **Text section and item name** | | **Page/line no(s).** | |
|  | | **info is located** | |
| **Title and abstract** | |  | |
| 1. **Title** | |  | |
| Indicate that the manuscript concerns an initiative to improve healthcare (broadly defined to include the quality, safety, effectiveness, patient-centredness, timeliness, cost, efficiency and equity of healthcare). | | Page 1, lines 1-2 | |
|  | |  | |
| 2. **Abstract** | |  | |
| a. Provide adequate information to aid in searching and indexing. | | Page 2, line 68. | |
| b. Summarise all key information from various sections of the text using the abstract format of the intended publication or a structured summary such as: background, local problem, methods, interventions, results, conclusions. | | Page 1, lines 34 - 36  Page 2, lines 37 -8. | |
|  | |  | |
| **Introduction: Why did you start?** | |  | |
| 3. **Problem description** - Nature and significance of the local problem. | | Page 3, lines 85 - 89 | |
| 4. **Available knowledge** - Summary of what is currently known about the problem, including relevant previous studies. | | Page 3, lines 90-102 | |
| 5. **Rationale** - Informal or formal frameworks, models, concepts and/or theories used to explain the problem, any reasons or assumptions that were used to develop the intervention(s) and reasons why the intervention(s) was expected to work | | Page 3, lines 97 – 108 | |
| 6. **Specific aims** - Purpose of the project and of this report. | | Page 4, lines 12 – 116 | |
|  | |  | |
| **Methods: What did you do?** | |  | |
| 7. **Context** - Contextual elements considered important at the outset of introducing the intervention(s). | | Page 5, lines 4-11. | |

| 8. **Intervention(s)** |  |
| --- | --- |
| a. Description of the intervention(s) in sufficient detail that others could reproduce it. | Pages 4, lines 141 -146 Page 5, lines 147 – 153 Page 6, lines 154 – 184 Page 7, lines 185 – 223 Page 8, lines 224 – 253. |
| b. Specifics of the team involved in the work. | Page 4, lines 128-137  Page 7, lines 189 – 191 |
| 9. **Study of the intervention(s)** |  |
| a. Approach chosen for assessing the impact of the intervention(s). | Page 7, lines 219 – 223  Page 8, lines 224 – 227 |
| b. Approach used to establish whether the observed outcomes were due to the intervention(s). | Page 7, lines 219 – 223  Page 8, lines 224 – 227 |
| 10. **Measures** |  |
| a. Measures chosen for studying processes and outcomes of the intervention(s), including rationale for choosing them, their operational definitions and their validity and reliability. | Page 6, lines 154 – 184 Page 7, lines 185 – 186, 213 – 217 |
| b. Description of the approach to the ongoing assessment of contextual elements that contributed to the success, failure, efficiency and cost. | Page 7, lines 213 – 217 |
| c. Methods employed for assessing completeness and accuracy of data. | Page 6, lines 178 -180. Page 7, lines 213 –223  Page 8, lines 224 – 227 |
| 11. **Analysis** |  |
| a. Qualitative and quantitative methods used to draw inferences from the data. | Page 6, lines 175 – 184 Page 7, lines 185 -186 Page 7, lines 205 – 211 |
| b. Methods for understanding variation within the data, including the effects of time as a variable. | Page 6, lines 117 -182. Page 7, lines 205 – 217. |
| 12. **Ethical considerations** - Ethical aspects of implementing and studying the intervention(s) and how they were addressed, including, but not limited to, formal ethics review and potential conflict(s) of interest. | As this study was considered a quality improvement initiate by the Expert Panel, no formal ethics review was advised, all survey data was anonymized and an independent person and group devised the evaluation survey, external to those involved in the development of these new methods. |
|  |  |
| **Results: What did you find?** |  |
| 13. **Results** |  |
| a. Initial steps of the intervention(s) and their evolution over time (eg, time-line diagram, flow chart or table), including modifications made to the intervention during the project. | Page 8, line 49 - 251 |
| b. Details of the process measures and outcomes. | Page 9, lines 271 – 273  Page 11, line 286 |
| c. Contextual elements that interacted with the intervention(s). | Page 8, lines 233 – 236 Page 245 245 – 253 Page 10, lines 283 – 285. |
| d. Observed associations between outcomes, interventions and relevant contextual elements. | Page 9, lines 271 – 273  Page 11, line 286 |
| e. Unintended consequences such as unexpected benefits, problems, failures or costs associated with the intervention(s). | Page 9, line 276 -278. Page 10, lines 280 -282. |
| f. Details about missing data. | Not applicable as there was no missing data. |
|  |  |
| **Discussion: What does it mean?** |  |
| 14. **Summary** |  |
| a. Key findings, including relevance to the rationale and specific aims. | Page 12, lines 289 – 294. |
| b. Particular strengths of the project. | Page 13, lines 352 -359. |
|  |  |
| 15. **Interpretation** |  |
| a. Nature of the association between the intervention(s) and the outcomes. | Page 12, lines 314-315, 19 -321. Page 13, lines 332 – 338. |
| b. Comparison of results with findings from other publications. | Page 12, lines 295 – 316. |
| c. Impact of the project on people and systems. | Pages 12, lines 314-316, 323-325. Page 13, lines 56 -359.  Page 14, lines 368 -371, 375-378. |
| d. Reasons for any differences between observed and anticipated outcomes, including the influence of context. | Page 13, lines 359-362. |
| e. Costs and strategic trade-offs, including opportunity costs. | Page 13, lines 360 -364.  Page 7, lines 189 – 191. Page 10, lines 283 – 285. |
|  |  |
| 16. **Limitations** |  |
| a. Limits to the generalisability of the work. | Page 13, lines 340 – 350 |
| b. Factors that might have limited internal validity such as confounding, bias or imprecision in the design, methods, measurement or analysis. | Page 13, line 364 Page 14, line 365 – 366 |
| c. Efforts made to minimise and adjust for limitations. | Page 13, lines 360 – 361 |
|  |  |
| **Conclusions** |  |
| a. Usefulness of the work. | Page 14, lines 383 – 390. |
| b. Sustainability. | Page 14, lines 383 -390. |
| c. Potential for spread to other contexts. | Page 14, lines 369 – 378 |
| d. Implications for practice and for further study in the field. | Page 14, lines 378 -378. |
| e. Suggested next steps. | Page 14, lines 380 -381. |
|  |  |
| **Other information** |  |
| 18. **Funding** - Sources of funding that supported this work. Role, if any, of the funding organisation in the design, implementation, interpretation and reporting. | Page 14, lines 485 – 488. |
|  |  |
|  |  |
|  |  |
|  |  |
| *Ogrinc G, et al. BMJ Qual Saf 2015;0:1–7. doi:10.1136/bmjqs-2015-004411* |  |
| *Downloaded from http://qualitysafety.bmj.com/ on January 2, 2017* |  |
