# Supplementary material for: Improving prioritization processes for clinical practice guidelines: new methods and an evaluation from the National Heart Foundation of Australia
Source: Health Res Policy Syst. 2023 Apr 5;21:26. doi: 10.1186/s12961-022-00953-9 (PMC10075165; doi:10.1186/s12961-022-00953-9)
Supplement: Supplementary file 2 — Additional file 2. Topics categorized using ICD-11 codes. Topics from public consultation extracted and categorized according to the International Classification of Diseases 11th revision, including topic keywords and frequency the topic was suggested in public consult responses. [file 12961_2022_953_MOESM2_ESM.docx]

**Additional file 2. Topics categorised using ICD-11 codes.**

| 1. Diseases of the circulatory system. (ICD Parent Code 11) | | |
| --- | --- | --- |
| Theme by ICD Code | **Topic keywords** | **Number of responses** |
| 1.1 Heart valve diseases   Mitral valve disease: BB60, BB61, BB62, BB63, BB64, BB65, NB31.40, BB6Y, BB6Z.   Aortic valve disease: BB70, BB71, BB72, BB73, BB74, BB7Y, BB7Z  Pulmonary valve disease: BB90, BB91, BB92, BB93, BB9Y, BB9Z, BC00, BC01, BC0Z, BC20 | Valvular heart disease (valve disease), aortic valve disease, valve replacement (TAVR), surgical aortic valve replacement (SAVR) Rheumatic heart disease (RHD, *Aboriginal and Torres Strait Islander peoples and RHD*) | 13 |
| 1.2 Structural developmental anomaly of heart or great vessels  LA80, LA81, LA82, LA83, LA84, LA85, LA86, LA87, LA88, LA89, LA8A, LA8B, LA8C, LA8D, LA8E, LA8F, LA8G, BE14.3, LA8Y, LA8Z, LA90, LA9Y, LA9Z. | Congenital heart disease, bicuspid aortic valve disease | 2 |
| [1.3 Heart failure](#one_two_HF)   BD10, BD11, BD12, BD13, BD14, KB40, BD1Y, BD1Z. | Heart failure (HF), environment and HF, congestive cardiac failure (CCF) | 16 |
| 1.4 Ischaemic heart disease and diseases of the coronary artery  BA40, BA41, BA42, BA43, BA4Z.  BA80, BA81, BA82, BA83, BA84, BA85, BA86, BE1A, BA8Y, BA8Z. | Coronary heart disease (CHD, acute and stable CHD) acute coronary syndrome (ACS), ischaemic heart disease (IDH), non-obstructive ACS, mental health and CHD, warning signs, *oral health and CHD,* environment and IHD, surgical vs PCI Myocardial infarction (MI or heart attack), *myocardial infarction in women (women’s heart attacks*), angina Medications (Dual antiplatelet therapy (DAPT) GpIIbIIIIa inhibitors, aspirin, statin, CHD and beta blockers, CHD and APA/statins)  Cardiac rehabilitation (cardiac rehab)  Coronary artery disease (CAD), spontaneous coronary artery dissection (SCAD), post-Kawasaki disease, *nutrition and CAD,*  Atherosclerosis, cardiac chest pain | 48 |
| 1.5 Cardiac arrythmia  BC60, BC61, BC62, BC63, BC64, BC65 | Atrial fibrillation (AF), rhythm disturbance, cardiac arrythmias, heart rate variability (HRV), arrythmia management,  Supraventricular tachycardia *(supraventricular tachycardia in women)* | 13 |
| 1.6 Hypertensive disease  BA00, BA01, BA02, BA03. BA04, KB45 | Essential hypertension, *hypertensive disorders of pregnancy,* post [surgical] hypotension | 25 |
| 1.7 Pulmonary heart diseases or diseases of pulmonary circulation  BB00, BB01, BB02, BB03, BB0Y, BB0Z. | Pulmonary hypertension | 1 |
| 1.8 Diseases of the myocardium or cardiac chambers  BC40, BC41, BC42, BC43, BC44, BC45, BC46, BC4Y, BC4Z | Hypertrophic cardiomyopathy,  Cardiomegaly (athletic heart syndrome, athlete’s heart) | 2 |
| 1.9 Cerebrovascular disease  8B00, 8B01, 8B02, 8B03, KA82, 8B0Z | Stroke, (stroke management*,* presenting signs of stroke*,* risk factors of stroke), *oral health and stroke* environment and stroke (climate change, air pollution at stroke) | 4 |
| 1.10 Diseases of the veins  BD70, BD71, BD72, BD73, BD74, BD75, JA05.7, JA61, BD7Y, BD7Z. | Venous thromboembolism (VTE and NOACs) | 1 |
| [1.11 Unspecified](#one_ten) | Cardiovascular disease (heart disease, CVD), inflammation, o*ral health and CVD (oral health and CHD, absolute risk, cardiovascular risk factor, heart health check, diabetes and heart disease (diabetes and CVD), diet and CVD,* environmental and CVD (climate change and CVD, air pollution and CVD)*, women’s heart health (women’s cardiac health), HRV detection, chronic disease impact on CVD,* upstream cardiovascular disease states | 37 |

| 1. Other diseases related to cardiovascular disease | |  |
| --- | --- | --- |
| Theme | **Topic keywords** | **Number of responses** |
| 2.1 Metabolic | Metabolic complications (metabolic problems), *hypercholesterolaemia*, cardiometabolic complications, lipids | 5 |
| 2.2 Urinary | Kidney disease, *management of renal disease,* | 2 |
| 2.3 Endocrine | Primary aldosteronism (*primary aldosteronism and hypertension),* type 2 diabetes (DM2 or T2DM, DM2 treatment), diabetes, Conn’s syndrome, *diabetes management, diabetes and heart disease (diabetes and CVD), obesity and diabetes* | 13 |
| 2.4 Oral Health | *Oral health and CVD (oral health and CHD)*, *oral health and stroke,* dental services, *periodontal disease* | 4 |
| 2.5 Mental health | *Anti-anxiety medication,* mental health impacts of disease, *anxiety in women* | 4 |
| 2.6 Unspecified | Chronic illnesses (chronic disease), chronic disabilities, *chronic disease impact on CVD* | 5 |

|  |  |
| --- | --- |

| 1. **Risk factors and prevention strategies** | |  |
| --- | --- | --- |
| **Theme** | **Topic keywords** | **Number of responses** |
| 3.1 Cholesterol | Cholesterol management, *hypercholesterolaemia* | 2 |
| 3.2 Physical activity | Exercise (lack or exercise, sedentary, active, lack of physical activity, incidentally active, exercise treatment protocols) | 9 |
| 3.3 Diet | Diet, (healthy diet, high carb, high sugar, processed food, vegetables, inflammatory foods, overeating, non-nutritious food, nutrition, low-carb diet, vegetables, full fat, alcohol), n*utrition and CAD, diet and CVD, s*ugar tax, healthy food supply | 19 |
| 3.4 Weight | Obesity (obese), overweight, *obesity and diabetes,* healthy weight, toxic fat | 10 |
| 3.5 Behaviours and prevention | Smoking (smoking cessation) | 2 |
| 3.6 Risk management | Risk factors (risk factor management), a*bsolute risk*, *cardiovascular risk factor,* risk assessment lipids, | 13 |
| 3.7 Built environments | Urban environments (town planning, public transport, designing cities, community gardens, society and the environment) | 2 |

| 1. General themes for guideline development | |  |
| --- | --- | --- |
| Theme | **Topic keywords** | **Number of responses** |
| 4.1 Support | Support (patient support and interventions, clinical support, evidence support, support and care, peer support group, decision support) | 3 |
| 4.2 Cardiac surgery or intervention | Surgical intervention, procedural intervention | 3 |
| 4.3 Cardiac devices | Cardiac devices | 3 |
| 4.4 Management | Patient management (maintenance, better management, self-management, patient self-care, self-monitor, self-manage, *lifestyle management,* action plans*),* adherence with therapy, complexities in management, disease management, long-term management, multi-disciplinary team care (MDT), hospital to community interface (transitions of care), *pharmacological management,* palliative cardiac care, shared decision making | 18 |
| 4.5 Prevention | Prevention (secondary prevention, preventative heart health) | 37 |
| 4.6 Patient behaviour change | Behaviour change (patient directed behaviour change)  Lifestyle (lifestyle choices, *lifestyle management,* lifestyle modification, lifestyle factors) | 12 |
| 4.7 Education | Education (health education, health literacy, education, information, resources), health promotion | 16 |
| 4.8 Pharmacological | Glucocorticoids (prednisone), mineralocorticoid (aldosterone), pharmacological therapy (pharmacological management), *anti-anxiety medication,* medications, medicines safety, deprescribing, polypharmacy, anticoagulants, prophylactic therapies | 14 |
| 4.9 Low vs. high value care | Low-value care vs. high-value care, risks of harm, potential benefits and costs, value to consumers | 4 |
|  |  |  |
| 4.9 Women | *Women’s heart health (women’s cardiac health),* *women’s heart attacks (MI in women)* heart disease in women, *anxiety in women, supraventricular tachycardia in women, hypertensive disorders of pregnancy (HDP),* women and inequity | 17 |
| 4.10 Indigenous Australians and Torres Strait Islanders | Indigenous Australians health (ATSI and health) climate change in remote Aboriginal communities, *Aboriginal and Torres Strait Islander peoples and RHD* | 13 |
| 4.11 Social determinants | Inequity in access, low income, affordability | 8 |
