# Supplementary material for: Improving prioritization processes for clinical practice guidelines: new methods and an evaluation from the National Heart Foundation of Australia
Source: Health Res Policy Syst. 2023 Apr 5;21:26. doi: 10.1186/s12961-022-00953-9 (PMC10075165; doi:10.1186/s12961-022-00953-9)
Supplement: Supplementary file 3 — Additional file 3. Matrix tool. This matrix tool with five domains was used by the Expert Committee during the consensus meeting to assist in prioritizing short-listed topics. [file 12961_2022_953_MOESM3_ESM.docx]

| Criteria | 1) Impact of disease | 2) Potential to impact health outcomes | 3) Organisation’s strategy | 4) Need from the organisation’s community | 5) Relevance to broad range of health care professionals | 6) Evidence base |
| --- | --- | --- | --- | --- | --- | --- |
| Consider | - Burden of disease, in terms of mortality, incidence or prevalence of disease  - Economic impact and costs | - Significantly improve health outcomes/promote health/reduce inequalities  - Significant or unexplained variation in clinical practice  - Feasibility of implementing a guideline  - Reduce avoidable mortality or morbidity | - Relevance to our 2018-2020 Strategy | -Topic representation during clinical themes public consultation by health care professionals and consumers  -Misconception about topic within the general community | - Relevance of topic to health care workers with a diverse level of expertise where most of the care is delivered by non-experts | - New, emerging or rapidly changing evidence or new care options - Complexity, controversy or uncertainty about topics and treatment - Level and quality of current evidence on topic  - Published guidelines or guidelines funded by our organisation within the past 2 years already exist |
| Ask | Does guideline development in this area have the potential to impact many people affected by this disease? | Would a guideline on this topic feasibly address or impact clinical practice and health outcomes and reduce variance in care? | Would a guideline on this topic be on strategy for the organisation? | Is there demonstrated feedback from our community that a guideline on this topic is needed? | Is care in this clinical area significantly delivered by non-experts? | Would a guideline on this have a strong evidence base or address any controversy in the interpretation of the current evidence base? |

Additional File 3. The matrix tool
