# Supplementary material for: Improving prioritization processes for clinical practice guidelines: new methods and an evaluation from the National Heart Foundation of Australia
Source: Health Res Policy Syst. 2023 Apr 5;21:26. doi: 10.1186/s12961-022-00953-9 (PMC10075165; doi:10.1186/s12961-022-00953-9)
Supplement: Supplementary file 4 — Additional file 4. Descriptive version of the matrix tool. Expanded version of the matrix tool listing considerations within each domain. This was provided to the Expert Committee before the consensus meeting. [file 12961_2022_953_MOESM4_ESM.docx]

**Additional File 4. Descriptive version of the matrix tool**

***Data supporting this criterion will be collected and summarised for each clinical guideline topic***

**Criterion 1**: **Impact of disease**
This criterion provides comparable data to assess the breadth of the impact of a disease.

This criterion covers separate components:

- Burden of disease, in terms of mortality, incidence or prevalence of disease (if possible, at different disease stages).
- Economic impact and cost, which includes direct costs such as hospitalisations, healthcare expenditure and indirect costs such as loss of income and productivity.

*This criterion essentially focusses on: Does guideline development in this area have the potential to* ***impact many people*** *affected by this disease?*

Considerations:

Burden of disease can be reported as ‘disability-adjusted life years’ (DALY). DALY is a measure of the years of healthy life lost from death and illness. It is the sum of years lived with disability (YLD), and years of life lost (YLL) due to premature mortality ^1^.

When assessing the economic impact of a disease, The World Health Organization^2^ recommends a prevalence-based approach is useful for understanding the current economic burden of disease, whereas an incidence-based approach is more suitable for understanding the future economic impact of a disease (and potential impact of prevention)^2^.

The World Health Organization^2^ recommends that the economic impact associated with a disease be considered at a macroeconomic and microeconomic level.

At a macroeconomic level, the aggregate impact across key economic determinants (non-health consumption, health status and leisure time) is established to provide a societal or economy-wide assessment. Factors include cost to society for medical expenses, impact on the Australia’s current gross domestic product and its future growth prospects.

At a microeconomic level, the economic impact on households, firms and government are considered. This includes the impact of ill-health from a disease on household income, savings or consumption patterns, household costs for medical expenses and impact on productivity at the workplace.

1. Australian Institute of Health and Welfare. Australian Burden of Disease Study Impact and causes of illness and death in Australia 2015. BOD 22. Canberra: Australian Institute of Health and Welfare. 2019.
2. World Health Organization. WHO guide to identifying the economic consequences of disease and injury, Geneva: World Health Organization. 2009.

***Data supporting this criterion will be collected and summarised for each clinical guideline topic***

**Criterion 2: Potential to impact health outcomes and reduce variance in care**
This criterion covers separate components:

- A guideline could significantly improve health outcomes/promote health/reduce inequalities
- Potential to reduce significant or unexplained variation in clinical practice including inappropriate care
- The feasibility of implementing a guideline within this area
- Guideline has the potential to reduce avoidable morbidity or mortality

*This criterion essentially focusses on: Would a guideline on this topic* ***feasibly address or impact clinical practice and health outcomes and reduce variance in care?***

Considerations:

Is there information available about sub-population groups who experience health inequalities when compared to the general population?

Is there unwarranted variance in the way best evidence is applied in clinical practice for this topic? Is there evidence of widespread inappropriate care?

Would reducing variance in clinical practice promote appropriate care or cost-efficiency?

Potential of guideline to reduce geographical variation in clinical practice and reduce inequity.

Potential to reduce harms and risks to patients and reduce overuse/underuse of tests and treatments, or those of low value.

***Data supporting this criterion will be collected and summarised for each clinical guideline topic* Criterion 3: Organisation’s strategy**

- Relevance to the organisation’s 2018-2020 Strategy.

*This criterion essentially focusses on: Would a guideline on this topic* ***be on strategy for the organisation?***

This parameter will also be considered during the clinical themes public consultation phase with input from the organisation’s internal departments including Development & Media, Marketing and Communications. ***Data supporting this criterion will be collected and summarised for each clinical guideline topic***

**Criterion 4: Need from the organisation’s community**This criterion covers separate components related to stakeholder and customer views:

- Topic representation during clinical themes public consultation by health care professionals and consumers
- Misconception about topic within the general community

*This criterion essentially focusses on: Is there demonstrated* ***feedback*** *from our community that a guideline on this topic* ***is needed?***

***Data supporting this criterion will be collected and summarised for each clinical guideline topic*Criterion 5: Relevance to broad range of health care professionals**

- Relevance of topic to health care workers with a diverse level of expertise where most of the care is delivered by non-experts.

*This criterion essentially focusses on: is care in this clinical area significantly delivered by* ***non-experts?***

Considerations:

Potential for guideline to impact clinical care delivered by health care professionals across all disciplines in topic area.

***Data supporting this criterion will be collected and summarised for each clinical guideline topic***

**Criterion 6: Evidence base**

This criterion covers separate components:

- New, emerging or rapidly changing evidence or new care options
- Complexity, controversy or uncertainty about topics and treatment
- Level and quality of current evidence on topic
- Guidelines published by the organisation within the past two years already exist
- Guidelines funded by the organisation within the past 2 years already exist

*This criterion essentially focusses on: Would a guideline on this theme have a* ***strong evidence base*** *or address any controversy in the interpretation of the current evidence base?*
