# Supplementary material for: Improving prioritization processes for clinical practice guidelines: new methods and an evaluation from the National Heart Foundation of Australia
Source: Health Res Policy Syst. 2023 Apr 5;21:26. doi: 10.1186/s12961-022-00953-9 (PMC10075165; doi:10.1186/s12961-022-00953-9)
Supplement: Supplementary file 5 — Additional file 5. 23-item online survey questionnaire. The 23-item online anonymized evaluation survey sent to Expert Committee members after the consensus meeting. [file 12961_2022_953_MOESM5_ESM.docx]

**Additional file 2. 23-item online survey questionnaire.**

| **Evaluation of Priority Setting Process survey** |
| --- |

**POPULATION PROFILE:**

16 members involved in priority setting process

**PRIVACY STATEMENT:**

The xxxxx, is collecting your survey responses for research purposes. Only the aggregated data will be used. Individual responses will not be personally identifiable.
 
Your Personal Information is being collected by the xxxxxxx to facilitate services requested by you and /or to keep you informed about our organisation’s related activities. We respect your privacy and embrace the principles contained in the Privacy Act. We may contact you in the future for the promotion of heart health messages and programs, research, fundraising purposes and invitations to events.  If you attend our events you may be photographed or filmed and images &/or audio may be used in various mediums to promote the organisation.   Please tell event staff if you do not wish to be photographed or filmed.  Personal details may be provided to third parties where required by law or for the purpose of facilitating services contracted by us, in so doing your personal information may be disclosed to overseas recipients.  Further information is available in our Privacy Notice or on request.  Communications from us may include mail, email, social media, SMS or telephone contact and may also include messages on behalf of event sponsors and other third parties. By providing your information to us you agree that you have provided your indefinite consent to this contact.  You may withdraw consent at any time though in doing so we may not be able to provide you with services requested. The Organisation will not disclose your information to any third party for their marketing purposes.
 
If you do not want to receive further communication from us (other than information that relates to this service) or if you have any questions about privacy please contact our Privacy Officer xxxxx.  Our APP privacy policy is set out in our Privacy Notice and details how you may complain about privacy issues and how we would deal with that complaint.  It also explains how you can access, correct or update information we hold about you. A copy of our Privacy Notice is available xxxxxxx

| **SECTION S: CLINICAL COMMITTEE MEMBER INFORMATION** |
| --- |

**INTRO:** Thanks very much for taking part in this important survey to share your thoughts about the xxxx Committee priority setting process.

First up, we’d like to know a little about you to get us started.

To begin the survey, simply click on the next button below.

<INSERT PRIVACY STATEMENT ON THIS INTRO PAGE>

| **MEMBERSHIP TYPE** (ASK ALL, SR, DNR)  S1. Firstly, which of the following best describes your role in the xxx Committee?  Expert committee member 1 Consumer representative 2 xxx staff member 3  Executive Subcommittee Member 4 |
| --- |

| **SECTION A: PUBLIC CONSULTATION** |
| --- |

**INTRO:** Thanks for that. First up we’d like to ask your thoughts on the **public consultation** part of the process for topic generation.

As a reminder, the public consultation process was open for seven weeks between November 2019 – January 2020 via an online survey, speaking to a wide cross section of our community, including researchers, health professionals and those living with heart disease.

A total of 450 topics were identified from 107 community’s responses, which were then reduced to a total of 127 unique topics.

| **PUBLIC CONSULTATION VALUABLE** (ASK ALL, SR, DNR)  A1. How **useful** do you think the public consultation was in being the first step in generating topics?  Very useful 1  Somewhat useful 2  Not very useful 3  Not at all useful 4 |
| --- |

| **OPEN RESPONSE RE-QA2** (ASK ALL, OPEN)  A2. For what reasons do you feel the public consultation was <INSERT RESPONSE FROM QA2>?  OPEN |
| --- |
| **QUALITY OF TOPICS GENERATED** (ASK ALL, SR, DNR)  A3. Do you feel that the topics generated through the public consultation were of…?  …very good quality 1  …good quality 2  …average quality 3  …poor quality 4  …very poor quality 5 |

| **CONTINUATION OF PUBLIC CONSULTATION** (ASK ALL, SR, DNR)  A4. And do you feel that the public consultation **should continue to be used** as the first step for the prioritisation of clinical guideline topic development?  Yes 1  No 2  Unsure 3 |
| --- |

| **REASON FOR CONTINUATION / NOT** (ASK ALL, OPEN)  A5. You mentioned you <do / do not> think the public consultation should continue to be used, why is that?  OPEN |
| --- |

| **SECTION B: THE MATRIX TOOL** |
| --- |

**INTRO:** Next up, we’d like to talk about the **matrix tool** used to prioritise the 5 short-listed topics.

As a reminder, following the public consultation, the 127 unique topics were categorized into 37 themes using ICD-11. From here, 32 of these themes were then excluded using an exclusion criteria under the guidance of the xxxl Committee Executive Group.

This resulted in the 5 topics short listed for the xxxx Committee priority setting meeting on March 18^th^, and as such used in the matrix tool.

Just to refresh your memory, an image of the matrix that was used for Ischemic Heart Disease is shown below:


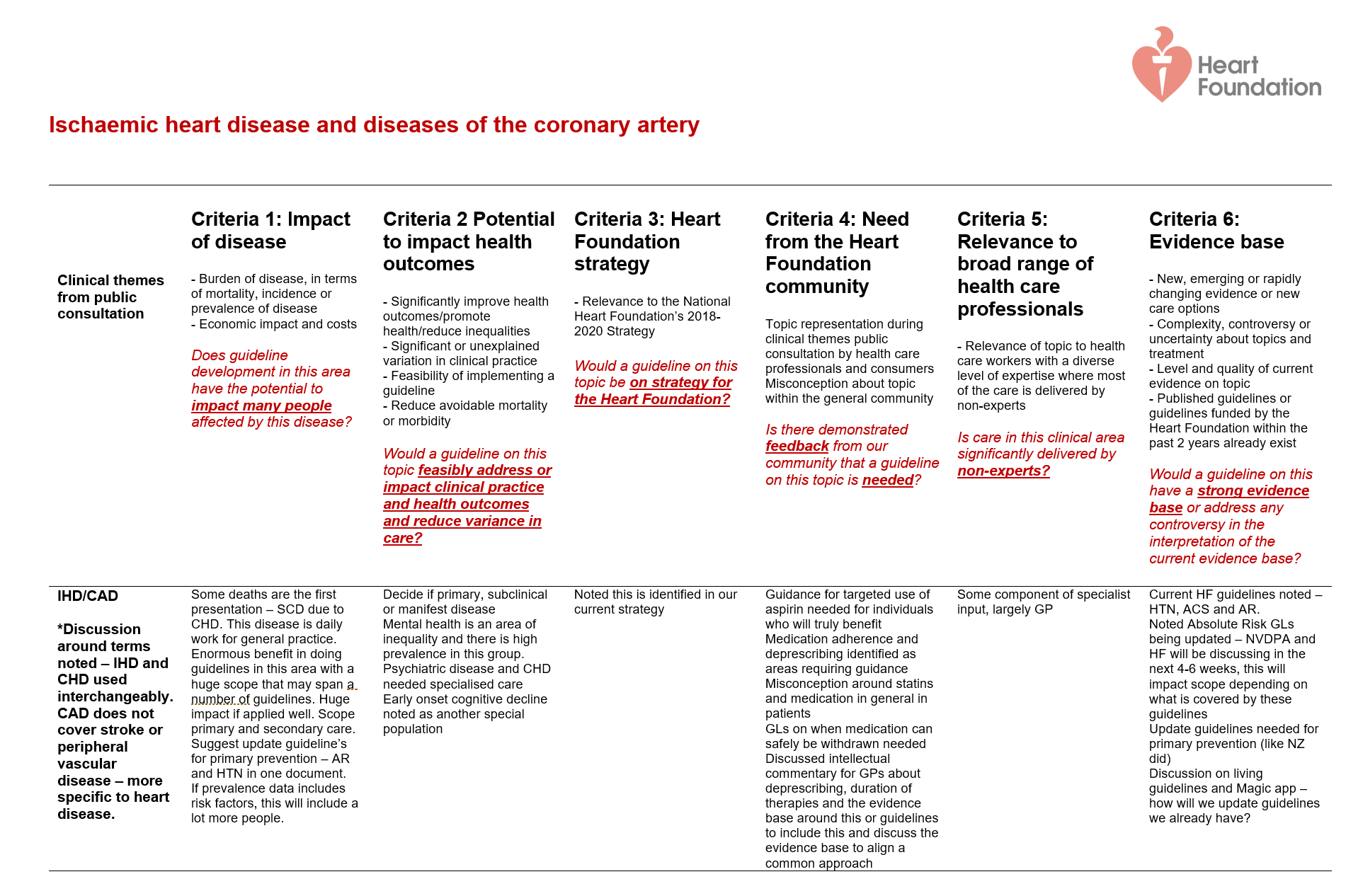


| **APPROPRIATENESS OF MATRIX TOOL** (ASK ALL, SR, DNR)  B1. Firstly, do you feel the matrix tool was **appropriate** to use in the prioritization of the 5 short-listed topics?  Very appropriate 1  Somewhat appropriate 2  Somewhat inappropriate 3  Very inappropriate 4 |
| --- |

| **REASON FOR APPROPRIATENESS** (ASK ALL, OPEN)  B2. You mentioned you felt the use of the matrix tool was <INSERT RESPONSE FROM ABOVE Q>. Why is that?  OPEN |
| --- |

| **MATRIX TOOL ASSISANCE WITH PROCESS** (ASK ALL, SR, DNR)  B3. Do you feel that the matrix tool **assisted** with the process of prioritising the 5 short-listed topics?  Assisted **a lot** 1  Assisted **a little** 2  Did **not really** assist 3  Did **not assist at all** 4 |
| --- |

| **MATRIX TOOL ATTITUDE STATEMENTS** (ASK ALL, SR PER ROW, RANDOMISE ROWS)  B4. To what extent do you agree that **the matrix tool**…?  ROWS  …contained all criteria required to make a considered judgement about each topic 1  …had an appropriate **number** of criteria (6) 2  …had **appropriate criteria** to consider for each topic 3  COLUMNS  Strongly disagree 1  Disagree 2  Neither 3  Agree 4  Strongly agree 5 |
| --- |

| **FEEDBACK ON MATRIX CRITERIA** (ASK IF DISAGREE WITH STATEMENT 3 ABOVE, OPEN)  B5. You mentioned you **did not** think that the matrix tool had **appropriate criteria** to consider for each topic. What criteria did you think was not appropriate? What did you think was missing?  *Please be as detailed as possible*  OPEN |
| --- |

| **SECTION C: PRIORITISAION PROCESS AS A WHOLE** |
| --- |

**INTRO:** Thanks very much for your responses so far! Now we’d now like to ask you a few questions about some technical aspects of the topic prioritisation videoconference held on xxxxx specifically.

| **F2F VS. VIDEOCONFERENCE** (ASK ALL, SR, RANDOMISE)  C1. As you will recall, the topic prioritisation meeting held on March 18^th^ was run via a video conference as opposed to a face to face meeting due to travel restrictions as a result of COVID-19.  If this type of meeting were to happen again in the future, would you suggest the meeting be run…  Via a videoconference again 1  As a face to face meeting 2  Other (Please specify:_______) 97 |
| --- |

| **ATTITUDES RE-RUNNING OF MEETING** (ASK ALL, SR PER ROW, RANDOMISE ROWS)  C2. Thinking again about the videoconference, to what extent do you agree with the following…?  ROWS  The purpose of the meeting was clear 1  The meeting was well chaired 2  The meeting ran for an acceptable duration 3  Participants felt able to share their opinions 4  COLUMNS  Strongly disagree 1  Disagree 2  Neither 3  Agree 4  Strongly agree 5 |
| --- |

| **ATTITUDES RE-PRESENTATION** (ASK ALL, SR PER ROW, RANDOMISE ROWS)  C3. And to what extent do you agree…?  ROWS  The presentation was well-structured 1  The presentation contained all necessary information to make informed decisions 2  The data presented was appropriate 3  The amount of data presented was just right 4  Rationale for decisions made were clearly articulated 5  The supporting documentation provided prior to the meeting was useful 6  COLUMNS  Strongly disagree 1  Disagree 2  Neither 3  Agree 4  Strongly agree 5 |
| --- |

| **MISSING CONTENT** (ASK ALL, OPEN)  C4. Did you feel there was anything missing from the videoconference presentation or documentation provided that would have been helpful to have? If so, what was missing?  OPEN  There was nothing missing 99 |
| --- |

| **VOTING ATTITUDE** (ASK ALL, SR, DNR)  C5. Thinking about the topic prioritisation videoconference held on March 18^th^ specifically, how **comfortable** were you with **verbally** ranking the topics in terms of their priority (as opposed to using an online voting tool)?  Very comfortable 1  Somewhat comfortable 2  Neither 3  Somewhat **un**comfortable 4  Very **un**comfortable 5 |
| --- |

| **ONLINE POLL PREFERENCE** (ASK ALL, SR, DNR)  C6. And regardless of how comfortable you were with verbally voting, would you prefer to use an **online voting tool** to rank the topics in terms of their priority in the future?  Yes 1  No 2  Do not mind either way 3 |
| --- |
| **ACCURACY IN PRIORITISING TOPICS** (ASK ALL, SR, DNR)  C7. Do you feel that the 5 topics discussed in the videoconference on March 18^th^ were **accurately prioritised** at the end of the process?  Yes 1  No 2 |

| **SECTION D: TECHNICAL QUESTIONS** |
| --- |

**INTRO:** Thanks so much, you’re nearly there! Finally, we just have a few remaining questions about your thoughts on the topic prioritisation process **as a whole**.

When we say the process as a whole, we want you to think about the process from the public consultation right through to reaching a consensus on the final 2 topics that were prioritised.

| **EFFECTIVENESS OF PROCESS** (ASK ALL, SR, DNR)  D1. Firstly, thinking about the topic priortisation process as a whole, how **effective** do you feel the whole process was in getting to the 2 final priortised topics?  Very effective 1  Somewhat effective 2  Neither 3  Somewhat ineffective 4  Very ineffective 5 |
| --- |

| **LIKES** (ASK ALL, OPEN)  D2. What, if anything, did you **like** about the topic prioritisation process as a whole?  OPEN |
| --- |

| **DISLIKES** (ASK ALL, OPEN)  D3. And what, if anything, did you **dislike** about the topic prioritisation process as a whole?  OPEN |
| --- |

| **CHANGES TO PROCESS** (ASK ALL, OPEN)  D4. Is there anything you would **change** about the process used to get to the 2 final prioritized topics?  *Please be as specific as you can about what you would change*  OPEN |
| --- |

| **WAS PROCESS SYSTEMATIC** (ASK ALL, SR, DNR)  D5. To what extent do you agree that the topic prioritisation process was **systematic** in its approach?  *Note: By systematic, we mean that the whole process was methodical or was done according to a clear plan*  Strongly disagree 1  Disagree 2  Neither 3  Agree 4  Strongly agree 5 |
| --- |

| **PROCESS TRANSPARENCY** (ASK ALL, SR, DNR)  D6. And To what extent do you agree that the topic prioritisation process was **transparent** in its approach?  *Note: By transparent, we mean that the whole process was done in such a way that it would be easy for others to see what actions were performed*  Strongly disagree 1  Disagree 2  Neither 3  Agree 4  Strongly agree 5 |
| --- |

| **PROCESS ATTITUDES** (ASK ALL, SR PER ROW, RANDOMISE ROWS)  D7. Again, thinking about the topic prioritisation process as a whole, to what extent do you agree that…?    The process…  ROWS  …followed a clear method 1  …was easy to follow through all stages 2  COLUMNS  Strongly disagree 1  Disagree 2  Neither 3  Agree 4  Strongly agree 5 |
| --- |

| **OPEN FEEDBACK** (ASK ALL, OPEN)  D8. Finally, do you have any further feedback about any aspect of the prioritization process we’ve spoken about today?  OPEN |
| --- |

**END SCREEN:** Those are all the questions for today. Thanks very much for having your say!
